# Fasting-Induced Natriuresis and SGLT: A New Hypothesis for an Old Enigma

**DOI:** 10.3389/fendo.2020.00217

**Published:** 2020-05-07

**Authors:** Samuel N. Heyman, Michael Bursztyn, Auryan Szalat, Mordechai Muszkat, Zaid Abassi

**Affiliations:** ^1^Department of Medicine, Hadassah Hebrew University Hospital, Mt. Scopus, Jerusalem, Israel; ^2^Department of Physiology and Biophysics, Ruth and Bruce Rappaport Faculty of Medicine, Technion-IIT, Haifa, Israel; ^3^Department of Laboratory Medicine, Rambam Health Care Campus, Haifa, Israel

**Keywords:** fasting, SGLT-2, hypertension, diabetes, obesity, glucose, sodium, natriuresis

## Abstract

For years, physicians and scientists were enthralled by the enigmatic phenomenon of fasting-associated diuresis and natriuresis and their reversal by feeding. This abrupt response is most prominent in obese and hypertensive individuals, and if repeated once and again may lead to the attenuation of blood pressure and improve insulin sensitivity. The mechanisms involved in early natriuresis and diuresis remain speculative as the renin–angiotensin–aldosterone axis and natriuretic peptides are initially suppressed. Based on gained insight using sodium–glucose transporter 2 (SGLT-2) inhibitors, herein, we propose a role for enhanced post-prandial proximal tubular sodium uptake, mediated by increased glucose–sodium co-transport, as daily filtered glucose increases, and reduced sodium uptake when glucose reabsorption diminishes. This phenomenon might be more pronounced in diabetics due to prolonged post-prandial hyperglycemia and intense SGLT-driven transport. Our hypothesis may also provide a physiologic basis for fasting-related reduced blood pressure in hypertension. This theory deserves challenging by experimental and clinical studies.

## Introduction

For five decades, physicians and scientists are enthralled by the enigmatic phenomenon of fasting-associated diuresis and natriuresis and their reversal by the administration of glucose (1, 2). These observations were mainly of clinical interest in the context of hospitalized patients for an intensive low caloric diet in the aim of losing weight (3). Interestingly, fasting natriuresis and carbohydrate-induced antidiuresis were noted to be especially prominent among hypertensive patients (4), and in spontaneously hypertensive rats (5), and develop quickly with natriuresis following an overnight fasting, with its abrupt reversal following glucose administration. Anecdotal layman reports of a desired 1-kg weight loss following fasting for a day (also reversed upon refeeding) likely reflect this phenomenon. Specific measurements in a fasting hospitalized patient could correlate exactly the amount of peak daily natriuresis (68 mEq) and diuresis/negative fluid balance (0.9 L) with loss of weight (0.9 kg), and the immediate reversal with carbohydrate intake (3).

Various mechanisms have been proposed to explain fasting natriuresis and diuresis and their reversal. As discussed below, insulin and the activation of the renin–angiotensin–aldosterone (RAAS) system have been suggested to enhance post-feeding sodium retention, while glucagon, natriuretic peptides, ketones, and the sympathetic system have been evaluated as mediators of fasting natriuresis. Yet, their role in these processes has not been conclusively established.

While no decisive explanation has been offered up to now, a new potential player in the involved mechanisms came to mind in light of the recent clinical trials and outstanding results related to the use of novel anti-diabetic oral medications of the sodium–glucose co-transporter 2 inhibitor (SGLT2-I) family.

SGLT2 inhibition is associated with enhanced natriuresis and diuresis, the consequence of diminished glucose–sodium co-transport in proximal tubules. These physiologic responses likely participate in the profound and very early reduction in cardiovascular morbidity and mortality in high-risk patients treated with SGLT2-I, in attenuating congestive heart failure and in the better control of hypertension (6). In the perspective of these outcomes with SGLT2-I, it is tempting to assume that alterations in sodium handling in proximal tubules, attributed to changes in filtered glucose via SGLT, might explain fasting diuresis and post-prandial sodium retention.

## Hypothesis

Glucose and sodium co-transport takes place at proximal tubular segments by the SGLT family, including SGLT1 and SGLT2 at the apical membrane and by GLUT 2 at the basolateral membrane. Fasting is associated with stable plasma glucose levels, provided initially by glycogenolysis, and later on by gluconeogenesis. Thus, glucose–sodium co-transport at the proximal tubule remains stable over time during fasting. In contrast, plasma glucose transiently increases following meals, with enhanced filtered glucose, leading to an increase in sodium–glucose co-transport. This is associated with an enhanced cortical oxygen consumption as the inhibition of SGLT improves cortical oxygenation (7). The following two hypothetical examples illustrate the postulated impact of meals on sodium tubular uptake. For the simplicity of the discussion, we ignore possible additional alterations in the glomerular filtration rate (GFR) and in sodium handling along the distal nephron segments.

### Example 1

**Figure 1:**
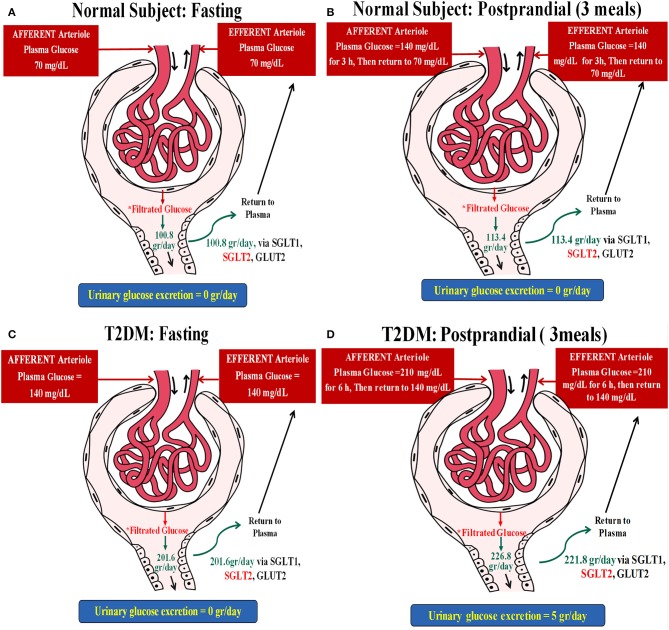
Two hypothetical examples illustrating the postulated impact of meals on glucose and concomitant sodium uptake in the proximal tubule. **(A)** A non-diabetic subject with stable glucose levels of 70 mg/dl and a glomerular filtration rate (GFR) of 100 ml/min. This individual will have a glucose reuptake of 70 mg/min (~100 g/day), as demonstrated below: Filtrated Glucose = PGluc × GFR = (70 mg/dl × 100 ml/min) × 24 h = (0.7 mg/ml × 100 ml/min) × 24 h = (70 mg/min) × 24 h = 70 ÷ 1,000 g/min × 1,440 min = 100.8 g/day. **(B)** This individual, now having three meals per day with three post-prandial increments of glucose levels to 140 mg for an hour will have an extra 12.6 g of glucose reabsorption per day: Filtrated Glucose = PGluc × eGFR = (70 mg/dl × 100 ml/min) × 21 h + (140 mg/dl × 100 ml/min) × 3 h = (0.7 mg/ml × 100 ml/min) × 21 h + (1.4 mg/ml × 100 ml/min) × 3 h = (70 ÷ 1,000 g/min × 1,260 min) + (140 ÷ 1,000 g/min × 180 min) = 88.2 + 25.2 = 113.4 g/day. Thus, ΔGlucose reabsorbed following meals = 113.4 – 100.8 = 12.6 g. **(C)** A type 2 diabetes mellitus (T2DM) patient with fasting glucose levels of 140 mg/dl without glycosuria and with a GFR of 100 ml/min will reabsorb 140 mg glucose/min, about 200 g/24 h while fasting: Filtrated Glucose = PGluc × eGFR = (140 mg/dl × 100 ml/min) × 24 h = (1.4 mg/ml × 100 ml/min) × 24 h = (140 mg/min) × 24 h = 140 ÷ 1,000 g/min × 1,440 min = 201.6 g/day. **(D)** Filtrated glucose in this patient post-prandial (three meals) = PGluc × eGFR = (140 mg/dl × 100 ml/min) × 18 h + (210 mg/dl × 100 ml/min) × 6 h = (1.4 mg/ml × 100 ml/min) × 18 h + (2.1 mg/ml × 100 ml/min) × 6 h = (140 ÷ 1,000 g/min × 1,080 min) + (210 ÷ 1,000 g/min × 360 min) = 151.2 + 75.6 = 226.8 g/day. Taking into account that this subject loses ~5 g of glucose per day, ΔGlucose reabsorbed = 226.8–201.6 = 25.2 g–5 g/day = 20.2 g/day. As proximal tubular transport of Na^+^ and glucose is matched with a 1:1 molar ratio for sodium–glucose co-transporter 2 (SGLT2), the non-diabetic individual (example 1), moving from a 24-h fast to regular eating, will have an additional daily uptake of 70 mEq Na^+^, while the diabetic patient (example 2) will have an enhanced sodium reuptake of 111 mEq. These amounts correspond to additional retentions of 4 and 6.5 g of sodium chloride per day for the non-diabetic and diabetic individuals, respectively. It translates to added water retentions of 0.5 and 0.66 L, respectively, in order to maintain extracellular osmolality, roughly the anecdotal reported acute weight reduction following fasting for a day, immediately reversed upon eating.

A non-diabetic patient with stable glucose levels of 70 mg/dl and a GFR of 100 ml/min will have a glucose reuptake of 70 mg/min, about 100 g/24 h. This individual, now having three meals per day with three post-prandial increments of glucose levels to 140 mg for an hour (additional 70 mg/min of filtered glucose for an hour, three times a day), will have an extra 12.6 g of glucose reabsorption per day (Figures 1A,B).

### Example 2

An obese individual with a poorly controlled type 2 diabetes mellitus (T2DM), fasting glucose levels of 140 mg/dl without glycosuria, and with a GFR of 100 ml/min will reabsorb 140 mg glucose/min, about 200 g/24 h while fasting. This patient will have protracted post-prandial hyperglycemia, for example, 210 mg/dl glucose for 6 h/day, an additional 70 mg/min glucose reuptake for 6 h, culminating with an extra 25.2 g of renal glucose handling per day. Excluding 5 g of glycosuria leaves us with an additional 20.2 g of daily tubular glucose reuptake as compared to fasting (Figures 1C,D).

As the proximal tubular transport of sodium and glucose is matched with a 1:1 molar ratio for SGLT2, the non-diabetic individual (example 1), moving from a 24-h fast to regular eating, will have an additional daily uptake of 70 mEq sodium, while the diabetic patient (example 2) will have an enhanced sodium reuptake of 111 mEq. These figures correspond to an additional retention of 4 and 6.5 g of sodium chloride per day for the non-diabetic and diabetic individuals, respectively. It translates to added water retentions of 0.5 and 0.66 L, respectively, in order to maintain extracellular osmolality, roughly the anecdotal reported acute weight reduction following fasting for a day, immediately reversed upon eating.

These figures are solely extrapolated from a plausible enhanced sodium transport by SGLT2. They are likely somewhat higher since some 10% of sodium–glucose co-transport is carried out by SGLT1 with a 2:1 transport molar ratio, respectively (8). Furthermore, an enhanced expression of co-transporters in the diabetic kidney minimizes glycosuria and intensifies sodium retention.

## Discussion

Our proposed hypothesis might explain the enigmatic phenomenon of fasting-induced diuresis and natriuresis in the absence of other reasonable established explanations. Our hypothesis may also shed light on the beneficial impact of fasting on resistant high blood pressure, with fasting for 2 days markedly attenuating hypertension in spontaneously hypertensive rats (5), while intermittent fasting without overall caloric deprivation reduces blood pressure in rats and humans (9, 10).

The mechanism(s) of fasting natriuresis have been thoroughly explored, mostly throughout 1970–2000, with no clear-cut explanations for this phenomenon. Noteworthy is that the mediators governing sodium and water handling may change over time, with the activation of physiologic responses to effective volume depletion following protracted fasting and sodium loss. Since fasting natriuresis develops rapidly, late neuroendocrine responses are likely secondary adaptive responses rather than causative factors.

Studying healthy individuals at baseline and following fasting for 5 days, Schloeder et al. (2) found that, after a huge water load (suppressing vasopressin), natriuresis was substantially enhanced in the fasting state as compared to a pre-fast period, an effect blunted within 20 min following glucose administration. Based on an increase in urine volume (*V*)/GFR and a decline in CH_2_0/*V* post-hydration, they concluded that fasting leads to a reduced sodium uptake by both proximal and distal tubular segments (2). This happens despite a profound increase in aldosterone secretion already at the early fasting phase (11).

Maoz et al. (12) reported that the atrial natriuretic peptide (ANP) levels doubled within 4 days in fasting obese hypertensive patients in parallel with urine sodium loss, suggesting a role for ANP in fasting natriuresis. Yet, a restricted diet in these patients was composed of 50% carbohydrates. Furthermore, in another study, obese patients with fasting natriuresis over 7 days showed no initial change in the atrial natriuretic factor, which even declined later on (13). In these experiments, a gradual increase in plasma aldosterone developed over time, without changes in renin and cortisol, altogether excluding a role for RAAS and natriuretic peptides in fasting natriuresis. In additional studies, a role for aldosterone in glucose-mediated sodium retention has also been ruled out (14). Interestingly, the elevation of aldosterone in the early phase of fasting, which seems adaptative to the fasting-associated enhanced natriuresis and volume contraction, is poorly antagonized by an anti-aldosterone medication such as spironolactone, suggesting that the distal tubular site of the reabsorption of sodium is not the main site involved in fasting natriuresis, neither in the anti-natriuretic effect of post-fasting refeeding, rather suggesting the involvement of the proximal tubular site (15).

Alterations in sympathetic activity have also been evaluated. Fasting for a few days with profound natriuresis occurred in obese normotensive patients, with no increase of sympathetic activity (16). Furthermore, the sympathetic system was not shown to play a role in sodium excretion in fed conscious rats. In contrast, fasting natriuresis, but not diuresis, has been facilitated by unilateral sympathectomy, as compared with the contralateral innervated kidney, while the GFR remained unchanged (17), suggesting a role for renal sympathetic innervation in the preservation of sodium during diet restriction.

Insulin receptors are found in the glomeruli and in most nephron segments, suggesting a physiologic role in sodium and water homeostasis in response to abrupt post-prandial water and osmotic loads. Insulin-clamp studies, maintaining a stable filtered load of glucose, suggest an independent effect of insulin, likely enhancing post-prandial sodium retention at the diluting segment of the distal nephron (18). Indeed, recent reports have shown that insulin exerts a RAAS-independent post-prandial reclamation of water and sodium, likely mediated by a significant increase in the open probability of ENaC along collecting tubules (19). Additionally, insulin also directly stimulates SGLT2 expression, as illustrated in studies using renal tubule-specific insulin receptor knockout (KO) mice (20). Yet, this likely does not explain the abrupt reversal of natriuresis following a short fasting period.

In contrast, glucagon was thought to participate in fasting natriuresis and refeeding antinatriuresis via anti-mineralocorticoid effects (11), but as exposed earlier, the involvement of aldosterone at the distal tubule does not seem to play a major role. A direct tubular effect of increased glucagon during fasting as the causative effect of fasting natriuresis and a reduced glucagon during carbohydrate refeeding as the cause of refeeding antinatriuresis has been ruled out, despite an indirect minor effect through ketogenesis being still possible (21). Indeed, an increased anionic ketone production mediated by reduced insulin and elevated glucagon levels during fasting is initially associated with an enhanced urinary sodium excretion to catch the urine excretion of the anionic ketone overproduction (3). Later on, the increased production of ammonium replaces the urinary sodium excretion, maintaining obligatory cation coverage of the metabolically generated anions as a major mechanism responsible for fasting natriuresis (22). Corroborating our initial hypothesis, and in view of the glucagon–ketogenesis pathway, the use of SGLT2-I was consistently associated with an increased risk of euglycemic ketoacidosis in treated diabetic patients (23), and it was recently shown that SGLT2-I in mice act on ketogenesis directly rather than *via* glucagon (24).

Otherwise, a plausible gut effect mediated by the kallikrein–kinin system following intragastric glucose administration has been shown to induce renal antidiuresis in anesthetized fasting rats. Yet, in these studies, natriuresis has been triggered by the administration of ANP (25).

Fasting natriuresis occurs despite enhanced Na/K/ATPase expression and activity within 24 h, both in the cortex and the medulla, as shown in psammomas (26). This shows that basolateral Na/K/ATPase is not involved in fasting natriuresis, which may, in part, reflect an adaptation to an enhanced sodium delivery to the distal nephron segments in response to a reduced proximal tubular sodium reabsorption.

Noteworthy is that, in addition to glucose, fructose, and sucrose also exert sodium retention in fasting natriuretic normotensive persons (27). Sucrose may do so following its hydrolysis with the release of glucose. Fructose is also reabsorbed in proximal tubules, coupled with sodium uptake at a 1:1 ratio. This transport is likely triggered by NH_3_-mediated (28) activation of SGLT4/5 and GLUT2 in the luminal and basolateral membranes, respectively (29).

Our theory ignores adaptive responses to a reduced proximal tubular sodium transport, affecting GFR and sodium and water handling along the nephron. Yet, such responses, triggered by the activation of the RAAS axis, are turned on following protracted fasting, when effective volume depletion becomes apparent, as experimentally shown in fasting rabbits (30). Furthermore, physiologic responses to SGLT2 inhibitors illustrate that distal tubular sodium reuptake does not fully compensate for sodium loss by the inhibition of proximal tubular glucose–sodium co-transport.

Our review ignores the possible additional effect of the different nutrition of lean and obese patients that could affect renal sodium and water handling through multiple mechanisms, such as the enhancement of GFR by amino acids, or diet-related modifications of renal transport mechanisms, for instance alterations in islet cell excretion of insulin and glucagon and the release of various gut peptides and adiponectin from adipose tissues. The latter, for instance, suppresses SGLT2 in proximal tubules. Thus, a decreased adiponectin in obese patients leads to an enhanced SGLT2 expression, likely governing salt retention and hypertension (31). Another example is the parallel upregulation of ion transporters and SGLT2 by a fructose-enriched diet (32).

As already mentioned above, our hypothesis takes into account the profound upregulation of SGLT2 in type 2 diabetics, likely enhancing post-prandial water and sodium retention. Additionally, other factors, not fully addressed here, are ensuing volume depletion due to osmotic diuresis, characteristic of uncontrolled diabetes and the non-ketotic hyperosmolar state, with the activation of the RAAS axis and renal sympathetic activity, reduction in GFR, and the likely inhibition of natriuretic peptides and the induction of diverse tubular sodium transporters (33–35). Such physiologic alterations may play in concert with post-prandial antidiuresis and salt retention, related to glucose–sodium co-transport, and counteract fasting-associated diuresis and natriuresis.

Abundant expression of natriuretic peptide clearance receptors (NPr-C) in the adipose tissues of obese patients attenuates diuresis in response to natriuretic peptides by reducing their accessibility to active receptors (36). Possibly, fasting could lead to a reduced NPr-C expression, facilitating natriuresis. Yet, most of the above-mentioned processes are likely retarded and do not explain early and abrupt diuresis upon fasting, immediately reversed by refeeding.

**Figure 2:**
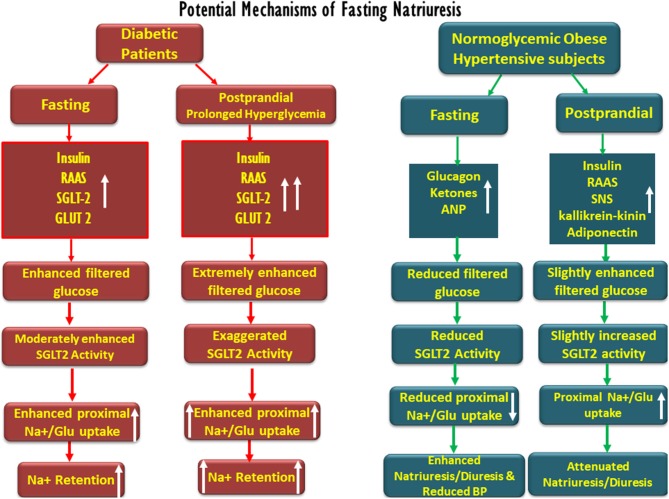
Schematic description of the potential factors involved in fasting-associated diuresis and natriuresis and their disappearance post-prandially in normoglycemic obese hypertensive subjects as compared with diabetic patients. Fasting natriuresis and post-prandial-induced antidiuresis are prominent especially among hypertensive patients. While glucagon, natriuretic peptides, and ketones have been suggested as mediators of fasting natriuresis, insulin and the activation of the renin–angiotensin–aldosterone (RAAS) system and the sympathetic nervous system (SNS) were suggested to enhance post-feeding sodium retention. At the tubular levels, fasting is associated with a reduced proximal tubular sodium uptake, mediated by an impaired SGLT-2 activity, whereas the opposite takes place post-prandially. The latter phenomenon might be more pronounced in diabetics due to prolonged post-prandial hyperglycemia and intense SGLT-driven transport.

Conclusively, although the applicability to human physiology of some of the findings shown in animals and reported above may be questionable, various mechanisms likely participate in extracellular sodium and volume homeostasis during intermittent feeding and fasting, as summarized in Figure 2. Yet, with no clear-cut evidence for the physiologic mechanisms fully explaining this phenomenon, early fasting diuresis and natriuresis, and post-prandial sodium and water retention may involve the attenuation and intensification of proximal tubular sodium transport, respectively, determined by tubular intraluminal glucose content. Our hypothesis may also provide a physiologic basis for fasting-related reduced blood pressure in hypertension. Fasting natriuresis may have a clinical relevance beyond being an interesting anecdote, and clinical studies are designed, assessing intermittent fasting as a potential therapeutic intervention in the management of the components of metabolic syndrome (10). Our proposed theory, therefore, deserves challenging by experimental and clinical studies. A reasonable approach might be the assessment of renal sodium and water handling when moving from fasting to feeding at baseline and in the presence of SGLT blockade. If proven correct, our hypothesis may form the theoretical basis for a broader clinical use of SGLT2 inhibitors, besides the management of T2DM.

## Data Availability Statement

The datasets generated for this study are available on request to the corresponding author.

## Author Contributions

SH, MB, and ZA proposed the new concept, participated in preparing the manuscript, and the figures. AS and MM participated in retrieving and elaborating on the relevant literature. SH, MB, AS, MM, and ZA wrote, read, and approved the final manuscript.

## Conflict of Interest

The authors declare that the research was conducted in the absence of any commercial or financial relationships that could be construed as a potential conflict of interest.

## Abbreviations

*V*: urine volume
GFR: glomerular filtration rate
CH_2_0: free water clearance
T2DM: type 2 diabetes mellitus
ANP: atrial natriuretic peptide
SGLT: sodium–glucose transporter.
